# Size-Dependent Optical Properties of InP Colloidal Quantum Dots

**DOI:** 10.1021/acs.nanolett.3c02630

**Published:** 2023-09-06

**Authors:** Guilherme Almeida, Lara van der Poll, Wiel H. Evers, Emma Szoboszlai, Sander J. W. Vonk, Freddy T. Rabouw, Arjan J. Houtepen

**Affiliations:** † Optoelectronic Materials Section, Faculty of Applied Sciences, Delft University of Technology, Van der Maasweg 9, 2629 HZ Delft, The Netherlands; ‡ Debye Institute for Nanomaterials Science, Utrecht University, Princetonplein 1, 3584 CC Utrecht, The Netherlands

**Keywords:** InP, quantum dots, phosphors, absorption, luminescence

## Abstract

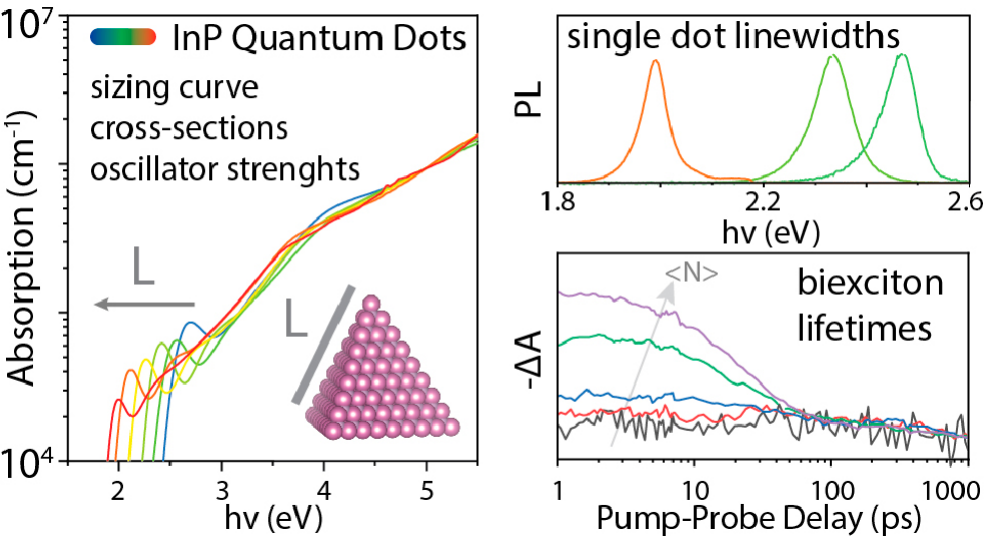

Indium phosphide colloidal quantum dots (CQDs) are the main alternative for toxic and restricted Cd based CQDs for lighting and display applications. Here we systematically report on the size-dependent optical absorption, ensemble, and single particle photoluminescence (PL) and biexciton lifetimes of core-only InP CQDs. This systematic study is enabled by improvements in the synthesis of InP CQDs to yield a broad size series of monodisperse core-only InP CQDs with narrow absorption and PL line width and significant PL quantum yield.

Indium phosphide colloidal quantum dots (InP CQDs) have raised considerable interest for photonic technologies operating in the visible and near-infrared regions because of their tunable band gap in the range of 1.3–3 eV, strong light absorption, efficient luminescence, and compliance with European safety regulations on electronic products (ROHS).^1^ However, the size-dependent optical properties of InP CQDs have not been reported to the level of detail that is common for other CQDs. This is due to difficulties in synthesizing luminescent samples of various sizes with narrow size distributions free of dopants (e.g., Zn) or shells (e.g., ZnSe_1–*x*_S_*x*_). In this regard, considerable progress has been recently achieved. On one hand, the works of Won et al., Li et al., and Xu et al. laid-down synthetic procedures to obtain monodisperse InP CQDs over a wide size range.^2−4^ On the other hand, recent works by some of the authors have shown that highly luminescent InP CQDs can be obtained by simply passivating their surface with In-based Z-type ligands, provided that the CQDs are oxide-free.^5^

By combining these methods, we prepare a size series of nearly monodisperse and bright InP CQDs with band gaps spanning a considerable portion of the visible spectrum and determine several fundamental size-dependent optical properties. First, we determine and quantify their sizing curves and light absorption characteristics by employing a combination of structural techniques and atomistic models. Radiative rates are extracted from photoluminescence (PL) transients recorded using time-correlated single-photon counting, and single dot PL line widths are measured by single-dot spectroscopy. Finally, power-dependent charge carrier recombination is investigated using transient absorption spectroscopy, which allows to extract biexciton lifetimes and Auger constants.

A size series of InP CQDs are prepared following reported procedures but conducting the syntheses under a hydrogen containing argon atmosphere of high purity in order to mitigate oxidation.^6^ While bulk InP has a band gap of ca. 1.33 eV, the InP CQDs studied in this work exhibit first-absorption-peak energies (*E*_1s_) in the range of 2.0–2.7 eV (620–460 nm) and thus cover a considerable portion of the visible spectrum. The narrow size distributions of these samples can be appreciated from the well-defined features in their absorbance spectra, shown in Figure 1a. The full width at half-maximum (FWHM) of the 1s exciton absorption peak decreases from 273 to 146 meV with increasing QD size.

**Figure 1:**
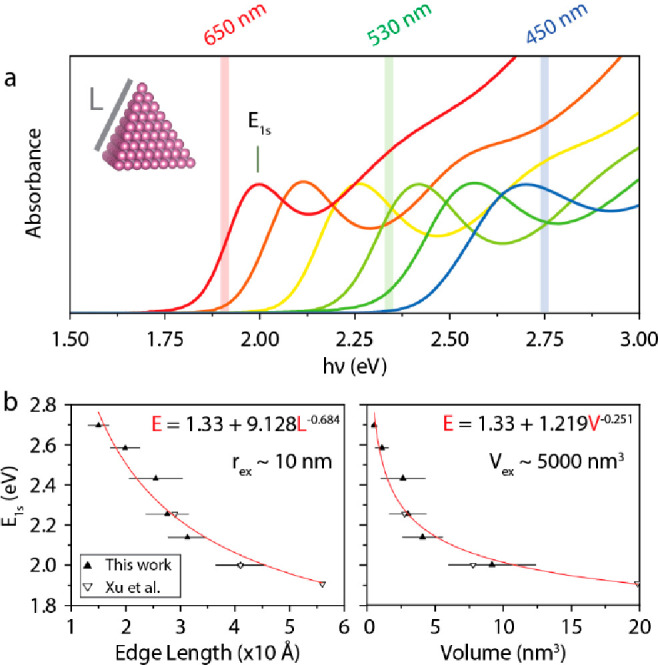
(a) Absorbance spectra of InP CQDs investigated in this work exhibit well-defined features characteristic of nearly monodisperse samples. Morphological analysis reveals that the QDs adopt a pyramidal shape and allows building (b) sizing curves relating the energy of the first absorption peak (*E*_1s_) with the average edge length and geometrical volume. It can be seen that these QDs are much smaller than the InP exciton Bohr radius (*r*_ex_) or volume (*V*_exc_): they are therefore in the strong quantum confinement regime.

InP QDs with band gaps in the visible range are relatively small when compared with other visible emitting QDs such as CdSe for instance, as shown in Figure S1 of the Supporting Information.^7^ This is not surprising because bulk InP has charge-carrier effective masses that are similar to those of CdSe but a band gap that is 400 meV narrower. The smallest QDs in our series have an edge length of 1.5 nm (corresponding to 5 In atoms) and a *E*_1s_ of 2.70 eV (460 nm). The smaller the QDs, the tighter the size distributions necessary for narrow ensemble line widths. On top of that QD single particle line widths also appear to broaden with decreasing size,^8−10^ and this is also the case for InP QDs as shown below. This partially explains the difficulties in obtaining InP CQDs with narrow emission line widths in the visible and makes the absorption spectra with distinct features particularly noteworthy.

We start by relating *E*_1s_ with the number-weighted average size and volume, determined from the analysis of electron micrographs shown in Figure S2. InP CQDs appear to adopt a (regular) tetrahedral shape, in line with previous reports,^4,11^ which allows their geometrical volume *V*_QD_ = *L*^3^/(6√2) to be determined analytically from their edge length *L*. Our InP CQDs exhibit edge lengths (volumes) in the range 1–4 nm (1–10 nm^3^). They are much smaller than the exciton Bohr radius (volume) which is ca. 10 nm (5000 nm^3^)^12^ and are therefore in the strong quantum confinement regime. The sizing curves are listed in Figure 1b. We have included extra data points from the work of Xu et al. for comparison and to extend the curves on the red side.^4^ It is found that *E*_1s_ ≈ 1.33 + 1.219*V*_QD_^–0.251^, with *E*_1s_ in eV and *V*_QD_ in nm^3^.

We also determine the energy of the second transition by taking the second derivative of the absorbance spectra and identifying the second negative peak. As shown in Figure S3, the energy of this transition and its separation from *E*_1s_ is also size-dependent.

Next, we quantify the absorption strength of InP QDs as a function of size, an important property for, e.g., phosphor applications. The (intrinsic) absorption coefficient α_*i*_ is first estimated experimentally through the Lambert–Beer law^13^
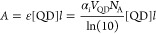
where *A* is the absorbance, ε is the extinction coefficient, *l* is the path length, and *N*_A_ is Avogadro’s number. The concentration of QDs, denoted [QD], is estimated as described in the Supporting Information. Briefly, it is estimated from the concentration of In atoms, measured via ICP-OES, assuming that the QDs are fully In-terminated and hence have a size-dependent stoichiometry (extracted from atomistic models). In addition, we consider volumes based on the discrete number of atoms, i.e., *V*_QD_ = *V*_In_*N*_In_ + *V*_P_*N*_P_ (the atomic radius of phosphorus (207 pm) is obtained from the InP unit cell, while that of indium (109 pm) is obtained from tabulated values).^14^ These atom-based volumes are slightly larger than geometrical volumes but more accurate as they fully account for surface atoms. Geometrical volumes do not account for surface atoms in full and introduce an important size dependent error, as shown in Figure S2f, due to the size-dependent fraction of surface atoms in QDs (see also Note 1 in the Supporting Information).

The experimental estimates of α_*i*_ (in heptane) are plotted in Figure 2a, alongside the bulk absorption coefficient α_b_. First, we note that α_*i*_ of InP CQDs is considerably smaller than α_b_ due to the dielectric screening of the electromagnetic field that occurs for small colloids in solution. These effects can be taken into account using effective medium approaches like the Maxwell–Garnett model.^13^ Therefore, we also calculate the absorption coefficient of a bulk-like InP colloid α_bc_, which, according to this model, is defined as
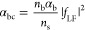
where *n*_b_ is the real part of the refractive index of bulk InP, *n*_s_ is the refractive index of the solvent (constant, 1.39 for heptane), and *f*_LF_ is is the local field factor, which in turn is defined as
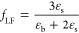
for spherical colloids, where ε_b_ is the complex dielectric function of bulk InP and ε_s_ that of the solvent (ε_s_ = *n*_s_^2^ for a transparent solvent such as heptane). Note that no analytical expression is available for tetrahedrons, but the effect of shape is likely small.

**Figure 2:**
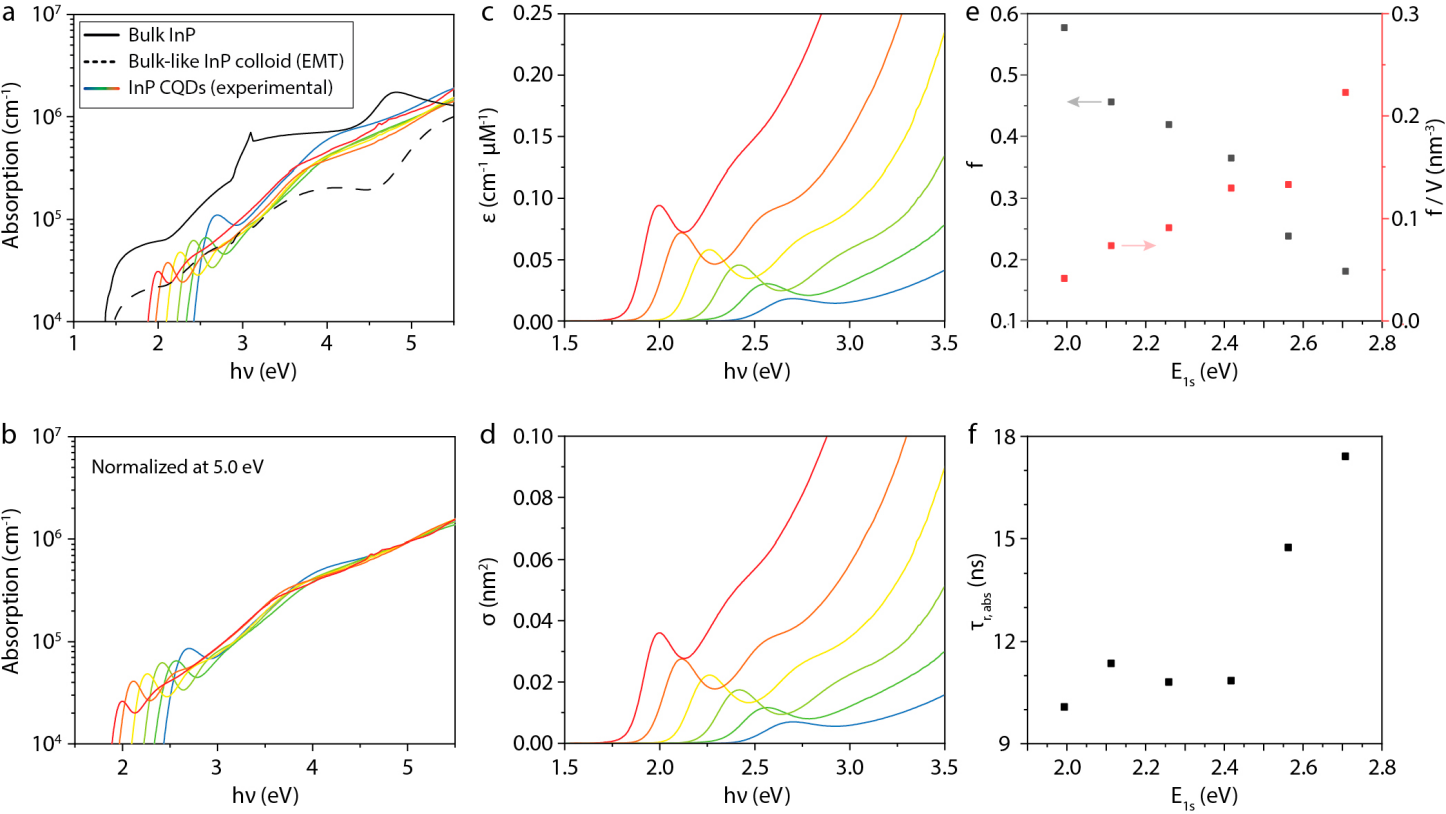
Absorptive properties of InP CQDs. (a) The experimental (intrinsic) absorption coefficient α_i_ of InP CQDs (in heptane) is plotted alongside that of bulk InP and that of a hypothetical bulk-like InP colloid (calculated following the effective medium theory model). Assuming that the absorption coefficients of the QDs converge at energies well above the gap, we (b) normalize the spectra to an average value at 5.0 eV. The (c) extinction coefficients and (d) cross sections are subsequently determined from the normalized α_*i*_ spectra. Finally, the (e) oscillator strength and the (f) lifetime of the band edge transitions are derived from the cross sections.

The estimated values of α_*i*_ of InP CQDs appear to be on the same order of magnitude as α_bc_ except for the 3.5–5 eV region. On one hand, we note that α_bc_ should only be taken as a rough guide as the optical constants of the QDs must differ from that of bulk and the InP QDs are not spherical. On the other hand, the deviation could also arise from the absorption of (metal) organic species or even InP clusters that might be present in the samples.^4,15^

Our estimates for α_*i*_ of InP CQDs follow the trend that is usually observed; i.e., they are strongly size dependent at the band edge but seem to converge at energies well above the gap.^13^ We assume that at high energies the minor deviation in α_*i*_ between samples is due to experimental uncertainties. Therefore, we rescale the α_*i*_ spectra such that the new value at 5.0 eV equals the average value in the six measurements (Figure 2b).

The extinction coefficient (ε = α_*i*_*V*_QD_*N*_A_/ln(10)) and the absorption cross section (σ = α_*i*_*V*_QD_) are subsequently determined from the rescaled α_*i*_ spectra and shown in Figures 2c and 2d, respectively. At *E*_1s,_ the extinction coefficient of InP QDs appears to be roughly half that of their equivolumetric CdSe counterparts, but at high energies (ca. 4 eV) it appears to be higher,^7,16^ similar to what is observed in bulk or for bulk-like colloids (see Figure S1).

The oscillator strength *f* of the band edge transitions is determined from the cross sections using the expression^17,18^
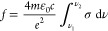
where ν_1_ and ν_2_ are the start and end frequencies of the band edge transition, *m* is the mass and *e* is the charge of an electron, ε_0_ is the permittivity of free space, and *c* is the speed of light. The cross sections were fit with Gaussian curves to extract the contribution of the band edge transition (Figure S4). The oscillator strength of the band edge transition is found to decrease with decreasing QD size (black data points in Figure 2e); however, when *f* is normalized by the QD volume, the opposite trend can be observed (red data points in Figure 2e), in line with the size dependence of α_*i*_ at the band edge (see Figure 2a).

Finally, the expected radiative lifetime (τ_r,abs_ = 1/*A*_*i*_) of the band edge transition can also be derived from the cross section through the Einstein coefficient (*A*_*i*_) defined as^17,18^
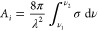
where λ is the wavelength of the optical transition. The computed radiative lifetimes of the band edge transition are plotted in Figure 2f: they are in the 10–20 ns range and increase with decreasing QD size. These computed radiative lifetimes are compared to measured PL lifetimes below.

We note that given its complexity, there is a significant error associated with the quantification of the absorptive properties. Such errors are, however, too difficult to determine given the many sources of error involved (weighing, dilutions, elemental quantification, size distributions, surface composition, etc.). We tried to minimize errors as much as possible (we believe the largest source of error arises from size, shape, and composition distributions), and the data presented herein are in line with expectations.

Photoluminescence (PL) transients of CQD ensembles are recorded using time-correlated single-photon counting and shown in Figure 3a. These PL transients are not single exponential but are well described by a biexponential function (fits are shown in Figure S6 and summarized in Table S1). The lifetimes of the main component (τ_1_) are in the range of 60–90 ns, while the weaker component exhibits lifetimes that are roughly twice longer (τ_2_ ≈ 150–200 ns). This yields amplitude-weighted average lifetimes (τ_av_) in the range of 80–120 ns.^19,20^

**Figure 3:**
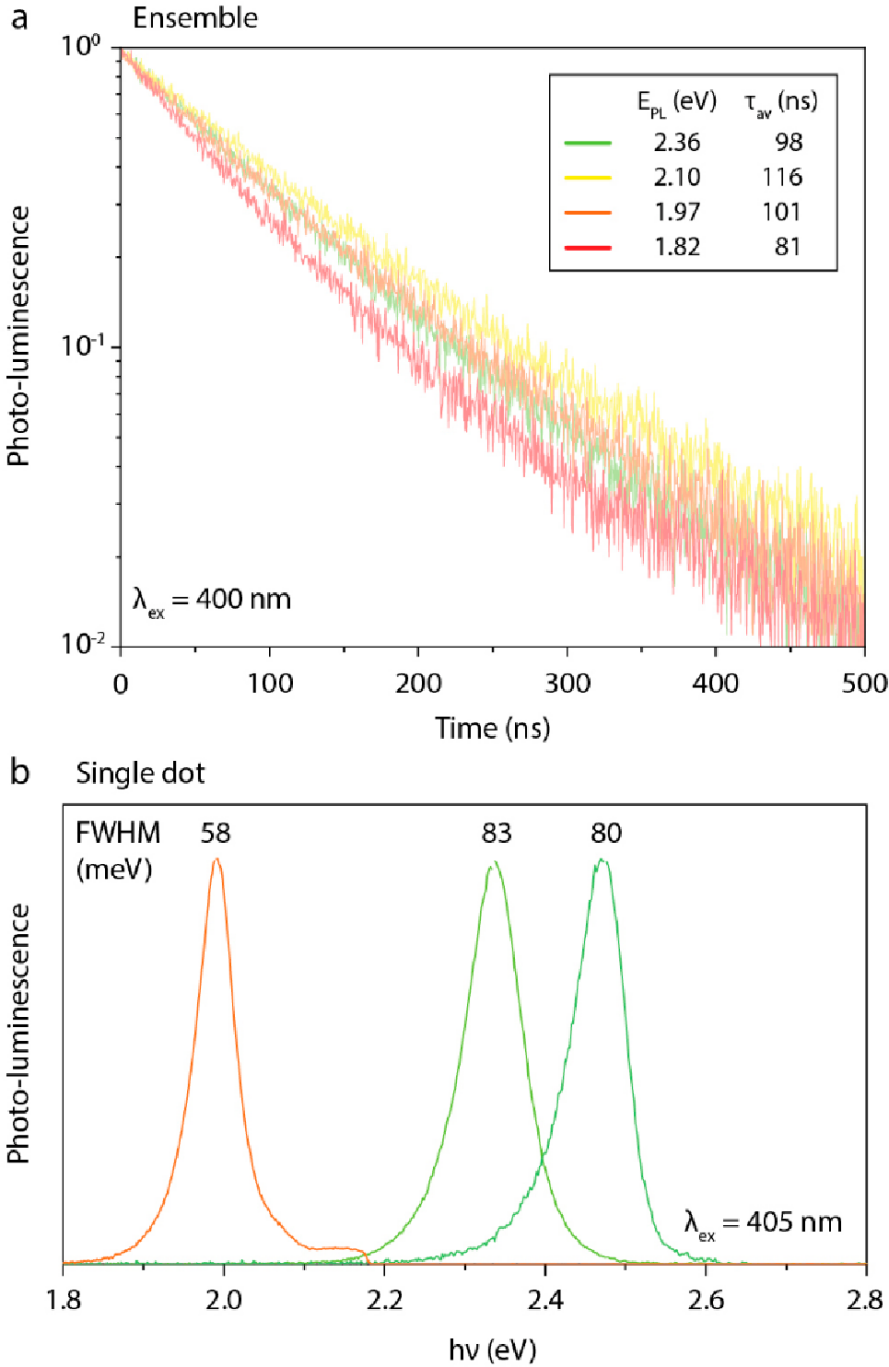
Luminescent properties of InP QDs. (a) PL decays of ensembles are recorded using time-correlated single-photon counting and are fit with biexponential decays with an amplitude average lifetime τ_av_. Photoluminescence quantum yields (Φ_PL_) are in the range 50–80%. (b) Single-dot PL spectra exhibit full widths at half-maximum (FWHM) of ca. 60 and 80 meV in the red and in the green, respectively.

Importantly, the expected radiative lifetimes calculated earlier from the absorption cross sections (τ_r,abs_, shown in Figure 2b) are 6 times shorter than those of τ_1_ and ca. 5–8 times shorter than τ_av_. This discrepancy can be explained by the fine structure of the band edge exciton.^21,22^ Briefly, in most semiconductors the ground exciton state is a dark state, and in small sized II–VI and III–V CQDs strong spatial confinement yields considerable splitting between the fine structure levels. The absorption is dominated by transitions to a bright exciton level with the highest oscillator strength. After excitation, relaxation brings the excited state to a thermal population over the lowest energy dark state and a bright state about several meV above it. The oscillator strength of this emissive bright state is lower than that of the absorbing bright state, and it is only partially populated at room temperature, which explains why the observed PL lifetime is longer than that calculated from the absorption coefficient. Similar conclusions have been drawn for InP/ZnSe_1–*x*_S_*x*_ core/shell CQDs.^23−25^ The results presented here suggest a similar fine structure for core-only InP QDs.

We also note that the PL lifetimes of red-emitting quasi-type I InP/ZnSe_1–*x*_S_*x*_ core/shell QDs with near unity quantum yields are considerably shorter, about 30 ns.^2,26^ However, it is unknown to what extent core-only and core–shell CQDs differ in fine structure.^27^ In addition, InP/ZnSe_1–*x*_S_*x*_ core/shell QDs have a different electron–hole overlap, with the electron wave function delocalizing into the shell, and hence also higher oscillator strengths given their much larger volumes. The fact that our InP core-only CQDs do not reach unity quantum yields also adds uncertainty to the analysis of the PL transients. On one hand, the PL transients most likely describe a sum of different transients from particles of varying QY.^28^ On the other hand, we cannot rule out the possibility that delayed luminescence may also contribute to the observed long PL lifetimes.

Next, single-dot PL spectra are measured using PL microscopy. First we note that these InP QDs exhibit blinking (Figure S8). Their PL spectra, shown in Figure 4b, exhibit full widths at half-maximum of ca. 60 and 80 meV in the red and in the green, respectively, which is considerably narrower than ensemble PL spectra (>200 meV in our samples). Both the values and the size-dependent trend are in good agreement with what is normally observed from single dots of other compositions.^8−10^ The single-dot spectrum is determined by the combined effect of phonon coupling and emission from multiple fine-structure states.^8^ In addition, we find that one of our single-QD measurements showed spectral diffusion of approximately 50 meV on a time scale of seconds (see Figure S8), which suggests that spectral diffusion might contribute significantly to line broadening.

**Figure 4:**
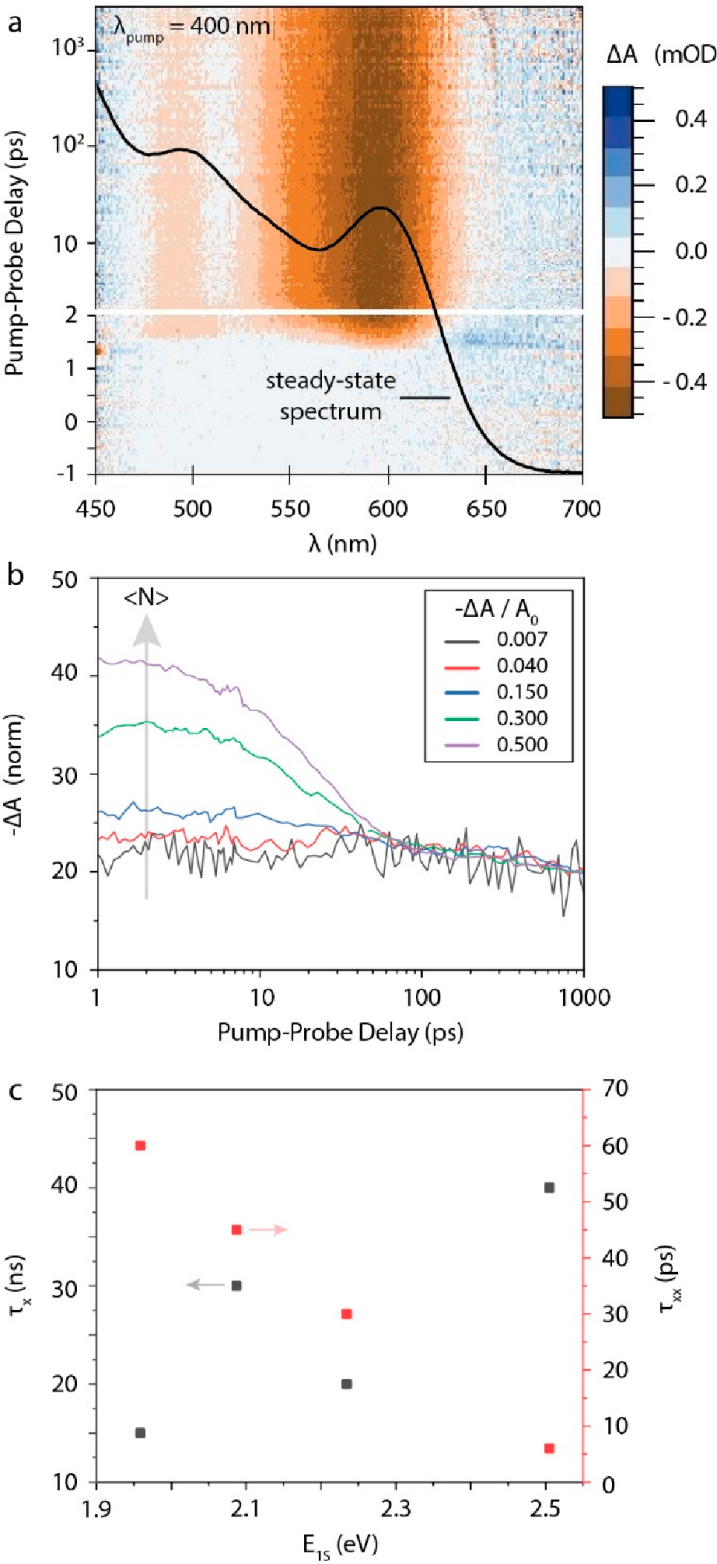
Transient absorption (TA) spectroscopy of InP CQDs. A typical TA spectrum is shown in (a). Kinetic analysis of (b) the band edge bleach at various pump powers (here defined by maximum value of – Δ*A*/*A*_0_) allows us to extract (c) the single-exciton (τ_*x*_) and the biexciton lifetimes (τ_*xx*_), and subsequently, to derive the Auger Constant for biexcitons (shown in the Supporting Information).

The relatively narrow single-QD line widths of 60–80 meV and much broader ensemble line widths (200 meV) are consistent with previous studies on InP-based core/shell QDs.^29^ Recent work has confirmed that InP-based core/shell QDs can have narrow single-QDs line widths but also showed that they on average suffer more from spectral diffusion (Figure S8b) and that inhomogeneous broadening is more significant than for CdSe-based QDs.^30^ Our measurements in Figure 3b show narrow single-QD line widths even for core-only InP QDs. This is encouraging, as it promises the possibility of narrower line widths in InP CQDs with improved surface passivation or engineered electron–phonon coupling.^8−10^

Finally, the kinetics of photogenerated charge carriers are investigated using power-dependent transient absorption (TA) spectroscopy. A typical lower power TA map, showing the change in absorption as a function of time and wavelength in false colors, is shown in Figure 4a (see Figure S9 for all TA maps). Briefly, exciting the samples at energies above the band gap quickly (<1 ps) leads to a bleach of the band edge (1S) transition. The decay of the band edge bleach is then analyzed as a function of pump power (see Figures 4b, S10, and S11). At low powers, the large majority of excited QDs only has one exciton, and the decays can be fit to a single-exponential decay with lifetime τ_*x*_ shown in Figure 4c. It can be seen that these single-exciton decays are in the order of 10–40 ns and appear to increase with QD size; however, because the maximum delay time of the measurement is 3 ns, these lifetimes are out of the measurement range and have a large associated uncertainty.

As the pump power is increased, so does the fraction of excited QDs with two or more excitons. This leads to the appearance of a fast component in the 10–100 ps time scale, as can be seen in Figure 3b, which is assigned to the fast Auger recombination. At intermediate powers, the decays can be described by adding a second exponential decay with lifetime τ_*xx*_, shown in Figure 4c. These biexciton lifetimes are quite short, in the range of 5–60 ps and correspond to Auger constants AC = *V*_QD_^2^/(8τ_*xx*_)^31^ in the range of (0.05–1) × 10^30^ cm^6^ s^–1^ (see Figure S12). The values are in line with previous measurements on InP/ZnSe_1–*x*_S_*x*_ core–shell QDs^2^ as well as with the universal size-dependent trend of Auger constants proposed by Robel et al.^32^ This shows that the scaling of Auger recombination in these small InP core-only QDs is similar to that of other QD materials.

Using recently developed protocols, we have synthesized a size series of monodisperse and highly luminescent InP core-only QDs. This series enables, for the first time, the systematic study of the size-dependent optical properties of core only InP QDs.

Based on absorption measurements, we report the size dependence of the band gap, second optical transition, absorption coefficient, oscillator strength, and associated radiative lifetime of the 1S absorption transition. Time resolved PL measurements yield PL lifetimes significantly longer than that expected from the absorption strength, showing that PL involves a different fine-structure level than the absorption. Single particle PL measurements reveal narrow single particle line widths of 60–80 meV at room temperature. Transient absorption measurement allows the determination of the size-dependent biexciton lifetime, which follows previously reported volume scaling trends for other QD materials.

Overall, the observed trends of the absorption coefficient, oscillator strength, and biexciton lifetime agree quite well with those obtained on other QD materials, after taking into account the bulk optical properties of InP and the generally smaller size of visible emitting InP CQDs when compared to CdSe CQDs.

## Supporting Information Available

Methods, morphology, and composition of InP CQDs, determination of the intrinsic absorption coefficient, sizing curves and absorption coefficients of InP and CdSe, determination of the second absorptive transition, fitting of the cross-section spectra, surface treatment of InP CQDs with In-based Z-type ligands, fitting of time-correlated single-photon counting transients, PL microscopy data, transient absorption spectra and their fitting, Auger constants (PDF)
