# Supplementary material for: Size-Dependent Optical Properties of InP Colloidal Quantum Dots
Source: Nano Lett. 2023 Sep 6;23(18):8697–703. doi: 10.1021/acs.nanolett.3c02630 (PMC10540257; doi:10.1021/acs.nanolett.3c02630)
Supplement: Supplementary file 1 — nl3c02630_si_001.pdf [file nl3c02630_si_001.pdf]

SUPPORTING INFORMATION FOR

Size-Dependent Optical Properties of InP Colloidal Quantum Dots

*Guilherme Almeida,[1] Lara van der Poll,[1] Wiel H. Evers,[1] Emma Szoboszlai,[1] Sander J. W. Vonk,[2] Freddy T. Rabouw[2] and Arjan J. Houtepen[1]**

[1]Optoelectronic Materials Section, Faculty of Applied Sciences, Delft University of Technology, Van der Maasweg 9, 2629 HZ Delft, The Netherlands.

[2]Debye Institute for Nanomaterials Science, Utrecht University, Princetonplein 1, 3584 CC, Utrecht, The Netherlands

Contents

0. Methods 2
1. InP vs. CdSe: sizing curves and absorption coefficients 4
2. Determination of the intrinsic absorption coefficient of CQDs..5
3. Morphology and Composition of InP CQDs 6
4. Determination of the second absorptive transition 7
5. Fitting of the cross section spectra..8
6. Surface treatment of InP QDs with In-based Z-type ligands..9
7. Time-Correlated Single Photon Counting 10
8. PL Microscopy..10

9. Transient Absorption spectroscopy 11

0. Methods

All synthetic procedures are executed in air-free conditions (Schlenk line or glovebox, H_2O < 0.1 ppm, O_2 < 0.1 ppm).

Materials. Palmitic acid (PA, 99%), myristic acid (99%), palmitic acid (99%), trioctylphosphine (TOP, 97%), heptane (99%, anhydrous) and acetone (99.8%, anhydrous) are purchased from Merck Sigma and used as received. Tris(trimethylsilyl)phosphine (98%, Strem), indium(III) fluoride (99.95%, anhydrous, Alfa Aesar), In(III) acetate (99.99%, anhydrous, Thermo Scientific), hexadecane (99%, anhydrous Thermo Scientific) are used as received. Octadecene (ODE, 90%, Merck Sigma) is degassed *in vacuo* at 100°C before being stored in a glovebox. Aqua regia is prepared using ultra-pure nitric and hydrochloric acids (Optima grade, Thermo Fisher). Argon (6N) and Argon (6N) mixed with 2% vol. Hydrogen (6N) are purchased from Linde.

Synthesis of InP CQDs. The smallest dots are synthesized following the procedure of Xu et al., intermediate ones through the method of Wu et al. and the largest ones according to that of Li et al.. Importantly, all the QDs are prepared under an ultra-pure argon – hydrogen atmosphere. At the end of the synthesis, the QDs are transferred inside an air-free glovebox, washed with acetone (three times) and re-dispersed in heptane.

In(III) Palmitate (0.2M) is prepared by heating a mixture of In(III) acetate, palmitic acid (3 eq.) and 1-octadecene in two stages. First under vacuum (<0.1 mbar) up to 100 °C and then at 150 °C under vigorous Ar (6N) bubbling for 30 minutes. The solution is then stored inside an air-free glovebox. We note that In-palmitate precipitates out of solution at room-temperature, and is therefore homogenized by heating (to ca. 100 °C) before use.

Surface Passivation with InX_3 is conducted according to previous reported protocols inside an air-free glovebox.[5] Briefly, InP CQDs (0.5 mL, 40 μM in dry hexadecane or 1-octadecene) are combined with InF_3 (50 mg, 0.29 mmol) and In(III) palmitate (100 μL of a 0.2 M solution) inside a glass vial (the amounts are indicative and were in fact adjusted slightly depending on the size of the QDs). The vial is tightly capped and the mixture is stirred at 150 °C for one hour. After cooling, InF_3 is separated out by centrifugation and the bright CQDs are washed with acetone and re-dispersed in heptane.

Transmission Electron Microscopy. Samples are drop cast onto grids and imaged on a JEOL JEM3200FSC microscope. The data is obtained using zero loss imaging (20eV) in counting mode and is movie corrected by SerialEM with a Gatan K2 summit camera.

Inductively Coupled Plasma Optical Emission Spectroscopy. Dry QD samples are digested in aqua regia (ca. 0.5 mL) inside a plastic tube), diluted with a HNO_3 solution (ca. 10 mL, 0.5 M, in ultra-pure milli-Q water) to achieve a concentration of In in the range of 1 ppm and analyzed using a Spectro Arcos spectrometer (detection range: 10 ppb to 20 ppm). The concentrations are determined against an external calibration.

Steady-State Absorption and Luminescence Spectroscopies. UV-Vis absorbance spectra are recorded on a Perkin-Elmer Lambda 365 spectrometer. Fluorescence measurements are recorded on an Edinburgh Instruments FLS980 spectrometer equipped with PMT detectors. For fluorescence measurements, the samples are loaded into air-tight cuvettes. Photo-luminescence quantum yields are measured in accordance with IUPAC methodology,[33] against a coumarin 102 dye solution in ethanol at an excitation wavelength of 387 nm. Measuring the coumarin 102 quantum yield in an integrating sphere in the same setup gave a value of 99%, but to calculate the quantum yield, the literature value of 95% is considered for the quantum yield of coumarin 102. [34]

Time-Correlated Single Photon Counting (TCSPC). Samples are loaded into air-tight cuvettes and photo-luminescence decays are collected on an Edinburgh Instruments Lifespec setup equipped a 400 nm pulsed laser. Amplitude-weighted lifetimes are calculated by the following equation: $\tau_{av}=(A_1\tau_1+ A_2\tau_2)/(A_1+ A_2)$ where A_n and τ_n are the n-th amplitude and lifetime parameters obtained from the bi-exponential fit.[19-20]

fs-Transient Absorption (TA) Spectroscopy . QD dispersions are loaded into air-tight cuvettes for TA measurements which are then conducted on a Light Conversion TA Setup. Briefly, an IR pulse (1028 nm, 180 fs) is producted by a Yb-KGW oscillator (Light Conversion, Pharos SP) at a frequency of 5 kHz. The pulse is split in two, one part is converted into a pump beam (wavelength tunable) by an optical parametric amplifier , while the other part is used to produce a broadband probe by supercontinuum generation in a sapphire crystal. Pump and probe beams overlap at the sample position with a small angle, with a relative time delay controlled by an automated delay-stage and the probe is then directed onto a photo-detector (Ultrafast Systems, Helios). During the experiments, we make sure the pump and probe beams have orthogonal polarizations (i.e. one of them is vertically polarized, the other horizontally), to reduce the influence of pump scattering into our detector. The pump beam is transmitted through a mechanical chopper operating at 2.5 kHz, allowing one in every two pump pulses to be transmitted. This allows to obtain pump-on and pump-off spectra, from which the differential absorbance $\Delta A = \log(I_{on}/I_{off})$ is determined (I is the probe light incident on the detector with either pump on or pump off). TA data are corrected for probe-chirp via a polynomial correction to the coherent artifact. Photon fluences are estimated by measuring the (pump) power with a thermopile sensor (Coherent, PS19Q) and taking into account the overlap of the pump and probe beams (imaged with a beamprofiler).

Photo-Luminescence Microscopy measurements are performed on a home-built optical setup consisting of a Nikon Ti-U inverted microscope body. A 405-nm diode laser (Picoquant D-C 405, controlled by Picoquant PDL 800-D laser driver) was guided to the sample by a dichroic mirror (edge at 425 nm, Thorlabs DMLP425R) and an oil-immersion objective (Nikon CFI Plan Apochromat Lambda 100x, NA 1.45). The QD emission was collected by the same objective and guided to a spectrometer (Andor Kymera 193i, 150 lines/mm reflective grating) with an electron-multiplying CCD detector (Andor iXon Ultra 888). To minimize photobleaching due to oxidation reactions, we deposit the nanocrystals on a glass coverslip inside a nitrogen-purged glovebox. We subsequently seal the QDs in between the coverslip and a microscope slide using an airtight spacer to ensure an oxygen-free environment during our measurements.

1. InP vs. CdSe: sizing curves and absorption coefficients

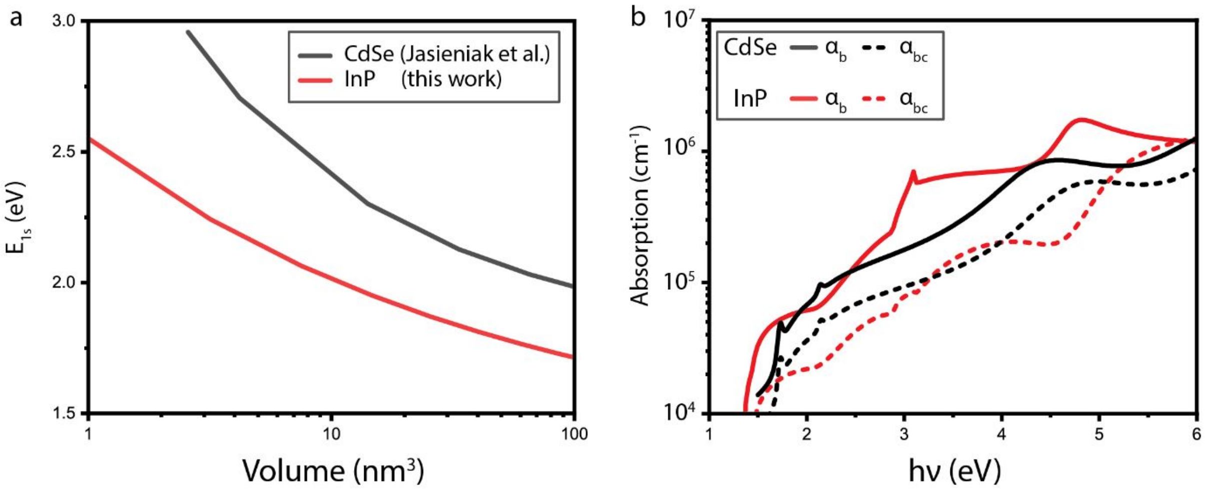

Figure S1 – (a) Sizing curve of tetrahedral InP compared to that of spherical CdSe CQDs (extracted from the work of Jasieniak et al.). (b) Absorption coefficients of bulk CdSe and InP (α_b) and their EMT-derived absorption coefficients for bulk like colloids (α_{bc}) in heptane (data for the zinc blende phase)

2. Determination of the intrinsic absorption coefficient of CQDs

The absorption coefficients α_i are estimated using the following formula

$$A = \frac{\alpha_i V_{QD} NA}{\ln(10)}[QD]l$$

where A is the absorbance, l is the path length, V_{QD} is the volume of a single QD, [QD] is the concentration of QDs and N_A is the Avogadro number.

In order to estimate α_i we determined the absorbance and the [QD] of concentrated “mother” dispersions in the following way:

a) CQDs are diluted in order to acquire absorbance spectra near the band edge, and diluted further to acquire absorbance spectra at higher energies. The dilution factors are carefully recorded (by mass) in order to retrieve the absorbance spectrum of the “mother” dispersion.

b) The [QD] is determined by dividing the [In] by the number of In atoms per QD which can be derived from the data shown in Figure S1d. The [In] is determined via ICP-OES. Briefly, a known volume of “mother” dispersion is dried, digested in ultra-pure *aqua regia* and diluted to a known volume before analysis (see Methods for further details). Again, the

volumes and dilution factors are carefully recorded (by mass) in order to retrieve the [In] in the mother dispersion.

We consider all QDs to be fully In-terminated (i.e. their composition is assumed to follow the fits shown Figures S1d and S1e) and employ atom-based volumes (derived from the fit shown in Figure S1f)

3. Morphology and Composition of InP CQDs

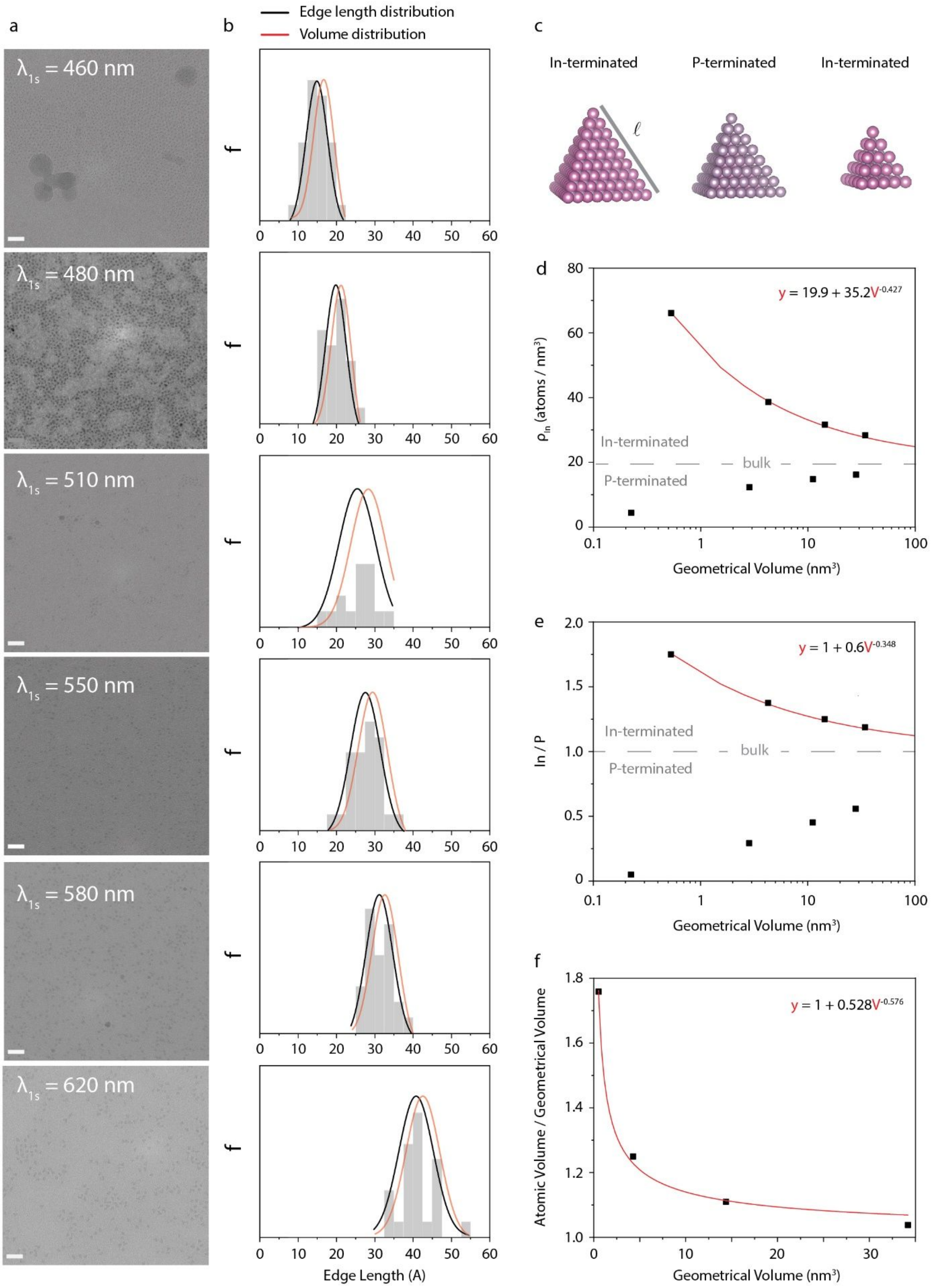

a
b
c
Edge length distribution
Volume distribution
λ_{1s} = 460 nm
λ_{1s} = 480 nm
λ_{1s} = 510 nm
λ_{1s} = 550 nm
λ_{1s} = 580 nm
λ_{1s} = 620 nm
f
Edge Length (A)
In-terminated
P-terminated
In-terminated
ℓ
d
ρ_{In} (atoms / nm^3)
y = 19.9 + 35.2V$^{-0.427}$
In-terminated
bulk
P-terminated
Geometrical Volume (nm^3)
e
In / P
y = 1 + 0.6V$^{-0.348}$
In-terminated
bulk
P-terminated
Geometrical Volume (nm^3)
f
Atomic Volume / Geometrical Volume
y = 1 + 0.528V$^{-0.576}$
Geometrical Volume (nm^3)

Figure S2-(a) Electron micrographs (scale bars 20 nm) and (b) size-distribution histograms of InP CQDs synthesized in this work. These CQDs are approximated to (111) terminated tetrahedral which can be either In or P terminated, depending on the size, as shown in panel (c). InP pyramidal models have therefore a size dependent composition which can be defined in terms of the (d) density of In atoms and the (e) In / P ratio as a function of volume. Note that at such small sizes the geometrical volume deviates, in a size-dependent fashion, from the volume obtained by summing the volume of individual atoms (we consider radii of 109 and 207 pm for Indium and Phosphorus, respectively), as shown in panel (f) for In-terminated QDs

Note 1: The absorption strength scales with the number of atoms. If the volume of a QD is measured from the center of a surface atom to the center of an atom on the opposite surface, this results in an underestimation of the number of atoms. Specifically, the surface atoms are not counted fully, which in turn would lead to an underestimation of the absorption strength.

4. Determination of the second absorptive transition

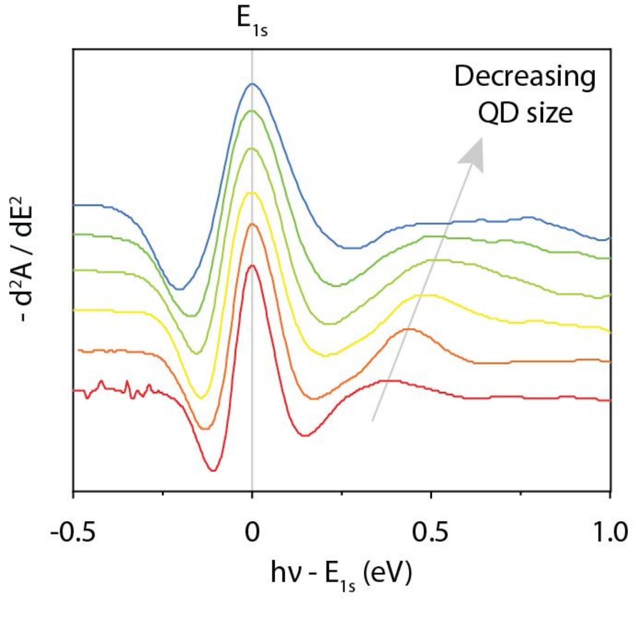

Figure S1 – Second derivative of the absorbance spectra reported to E_{1s}. It can be see that energy separating the E_{1s} from the second transition is size-dependent, i.e. it increases with decreasing size, like E_{1s}.The separation energy $\Delta E \approx 0.41 E_{1s}$ -0.44, with ΔE and E_{1s} in eV.

5. Fitting of the cross section spectra

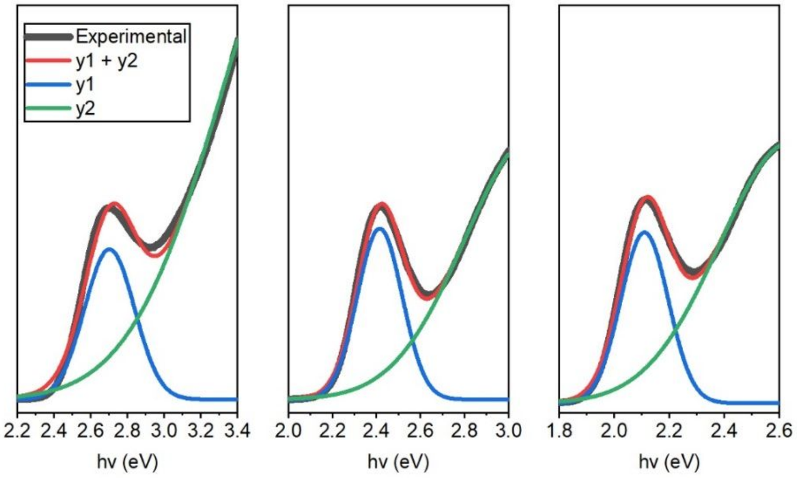

Figure S2 – By fitting the cross section spectra with two Gaussian functions (denoted y1 and y2) the band edge transition (y1) is extracted and subsequently integrated in order to determine its oscillator strength and lifetime.

6. Surface treatment of InP QDs with In-based Z-type ligands

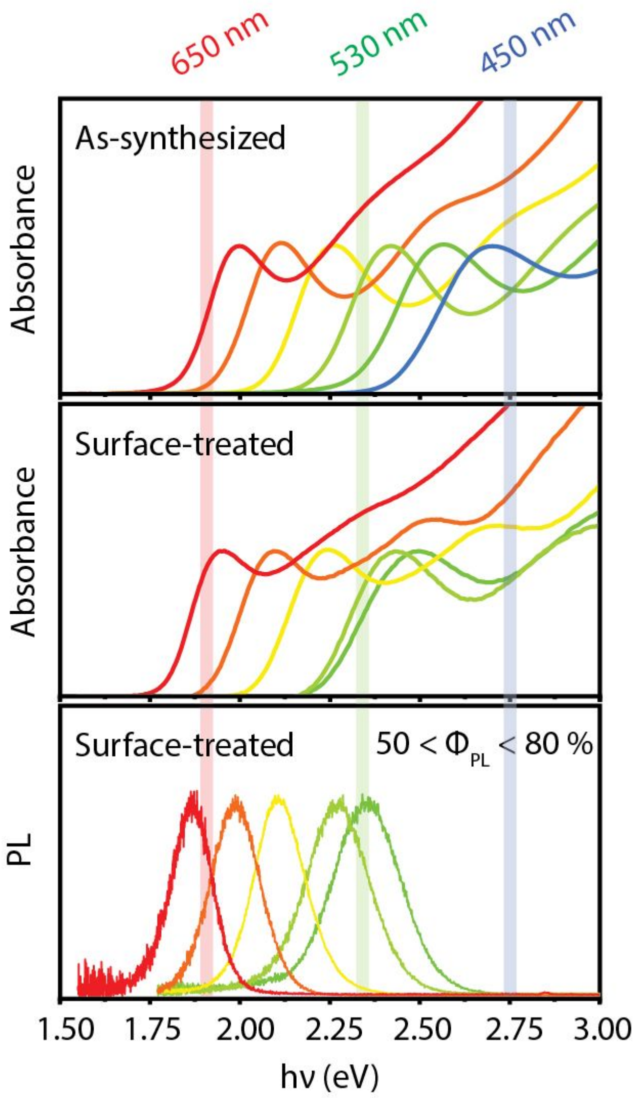

Figure S3 – Treating the surface of our InP CQDs with a mixture of InF_3 and In(III) palmitate boosts their photo-luminescence quantum yields to 60-80% but also leads to small changes in their absorbance spectrum indicative of a broadened size distribution and of small changes in average size (the smallest dots exhibited a red-shifted PL, in the green, and therefore we opted not to include them for clarity).

7. Time-Correlated Single Photon Counting

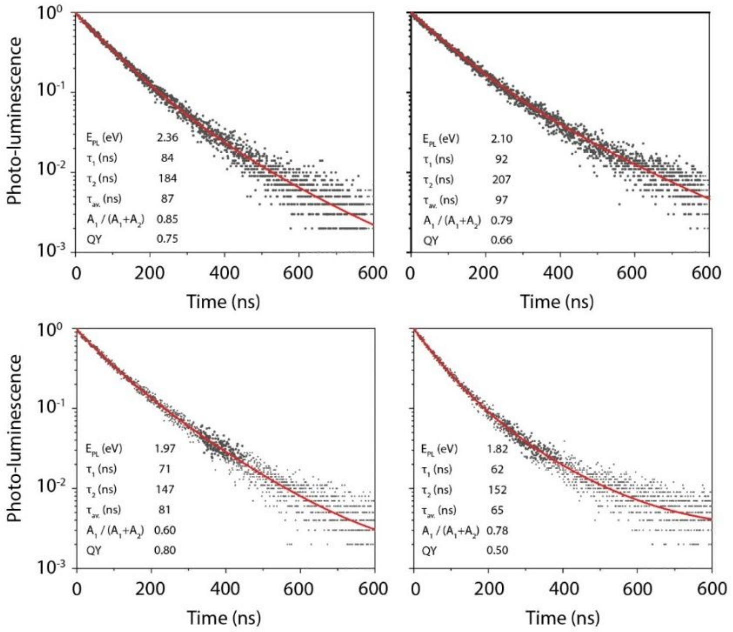

Figure S6 – Photo-luminescence transients of ensembles are recorded using time-correlated single-photon counting and are fit with bi-exponential decays with time constants τ_1 and τ_1 and amplitudes A_1 and A_2. Photo-luminescence quantum yields (Φ_{PL}) are in the range of 50 to 80%.

Table S1 – Summary of PL transient analysis

E_{PL} (eV)	τ_1 (ns)	τ_2 (ns)	$A_1/(A_1+A_2)$	τ_{av} (ns)	Φ_{PL}
2.36	84	184	0.86	98	0.75
2.10	92	207	0.79	116	0.66
1.97	71	147	0.60	101	0.80
1.82	62	152	0.79	81	0.50

8. PL Microscopy

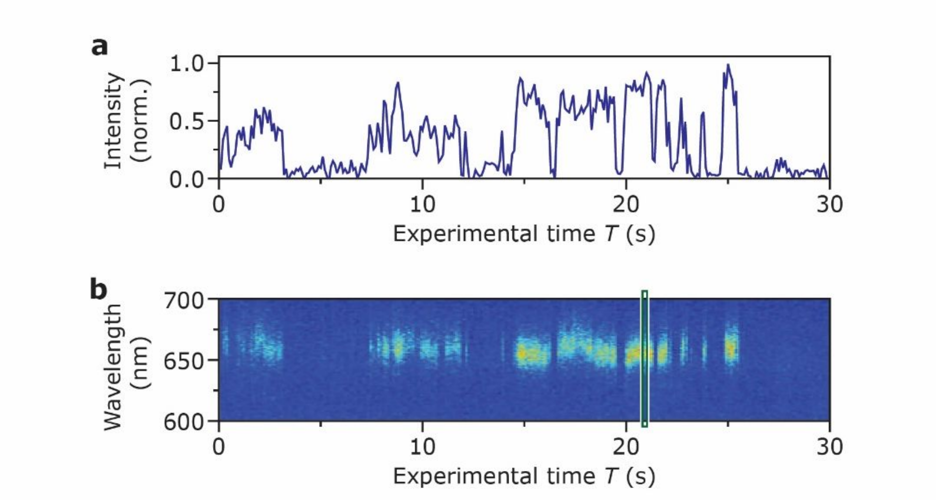

Figure S8 – (a) Intensity trace of a single InP QD with 100-ms time bins shows switching between a bright ON and a dark OFF state. (b) Spectral trace of the same measurement showing significant spectral diffusion on the second timescale.

9. Transient Absorption spectroscopy

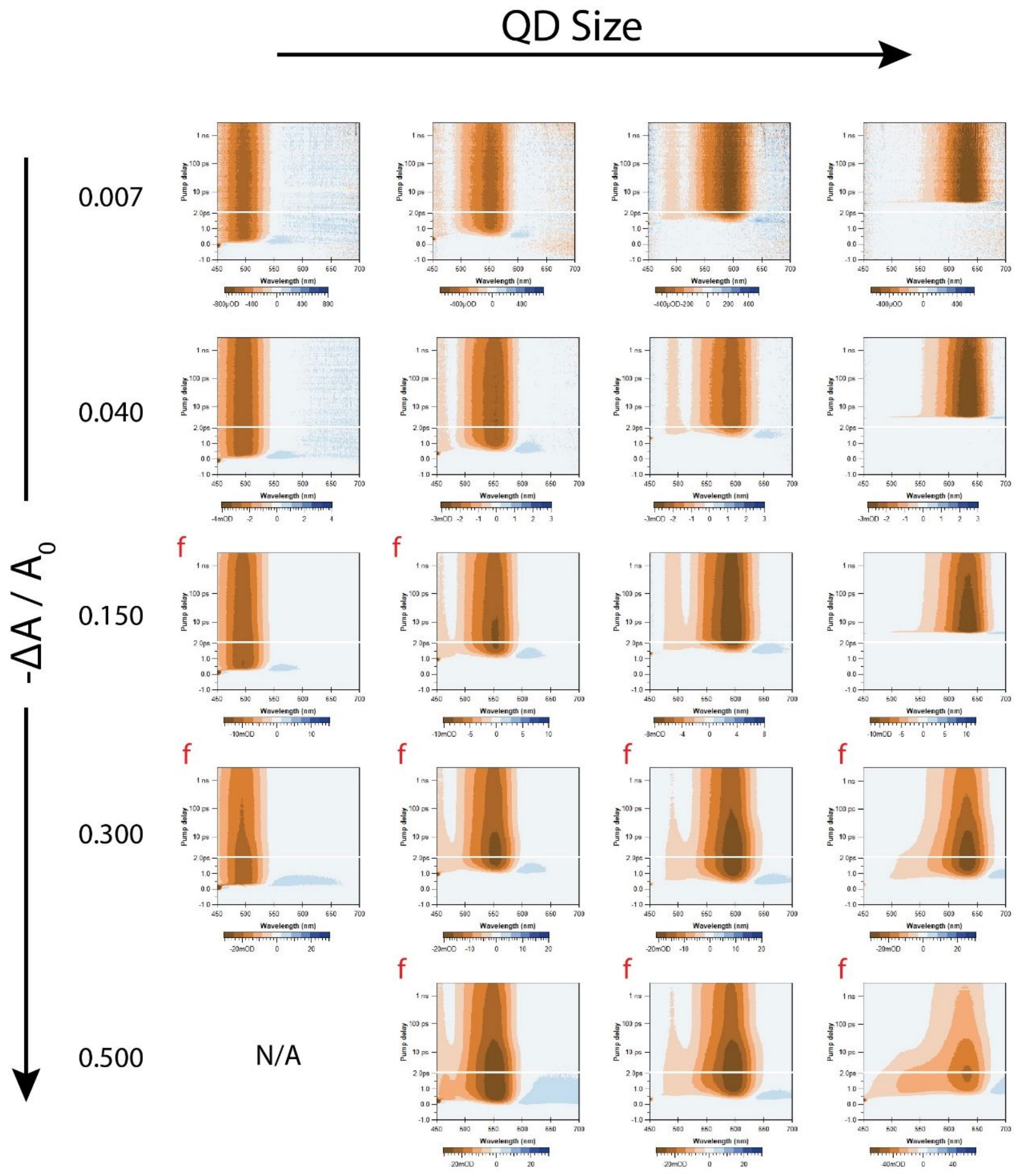

Figure S9 – Transient Absorption spectra of InP CQDs as a function of size and pump-power (or $\Delta A/A_0$). Spectra labelled with a red "f" were acquired using a focused pump. In these conditions the pump diameter becomes similar to the probe diameter which complicates their interpretation, therefore we did not employ them for kinetic fittings.

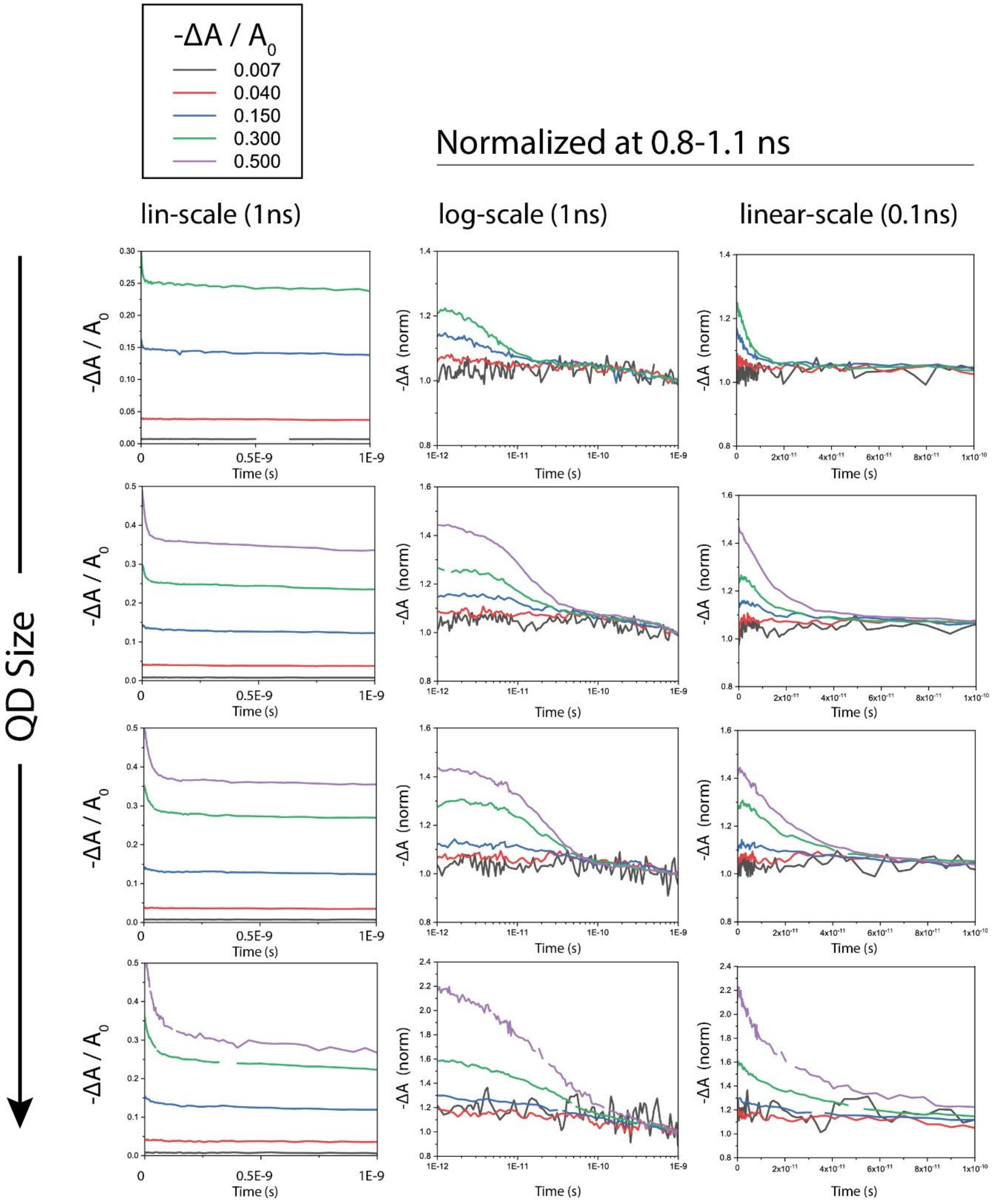

Figure S10 – Transient Absorption Decays integrated over the 1s band as a function of QD size and pump-power (or $\Delta A/A_0$).

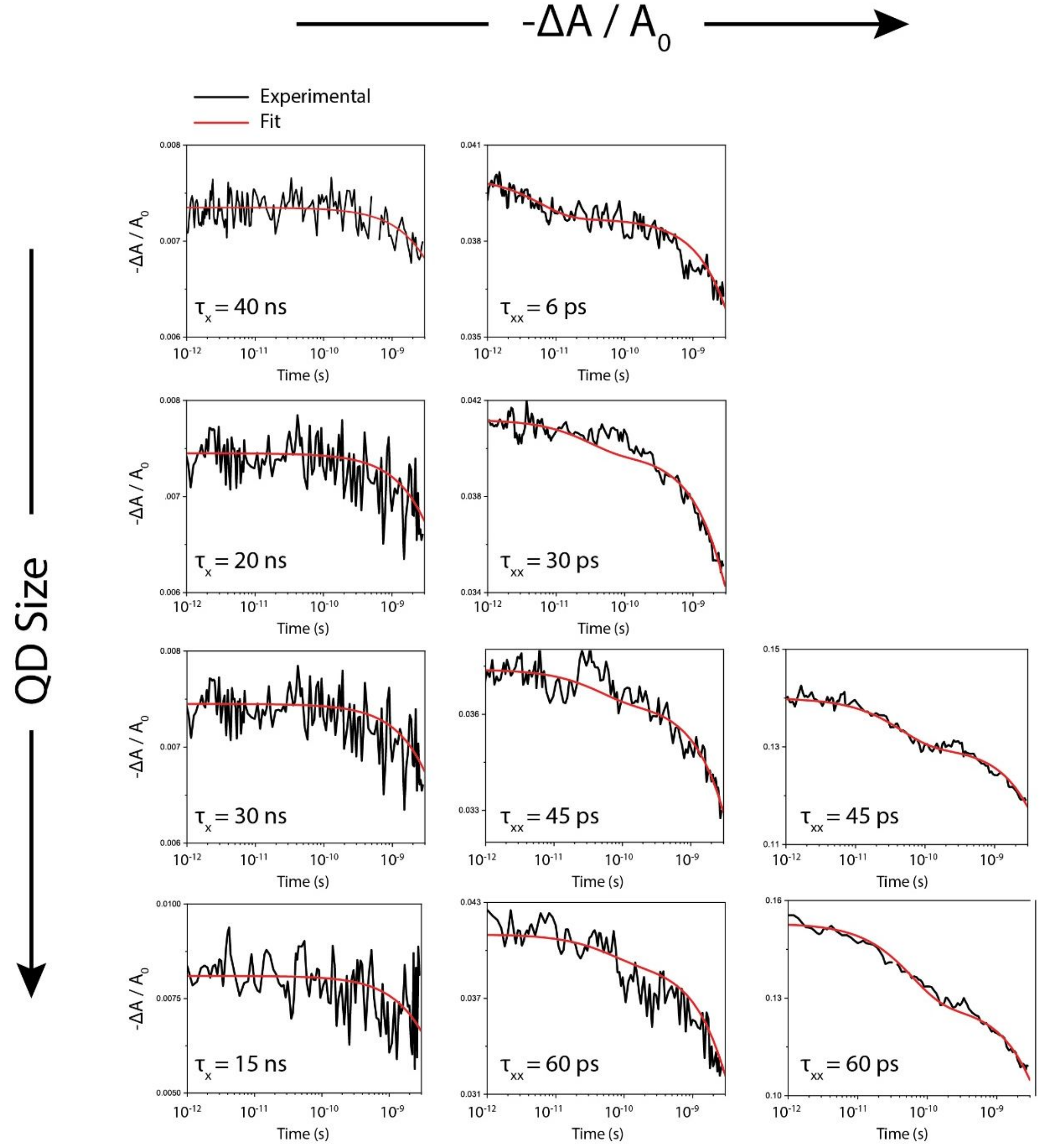

Figure S11 – Fitting of the TA decays. At low fluences the spectra can be fit with a single exponential decay. At slightly higher fluences an additional faster component arises due to a fraction of bi-excitons and these spectra were fit with a bi-exponential (neglecting the formation of single excitons from the decay of biexcitons, since the relative amount of biexcitons is small).

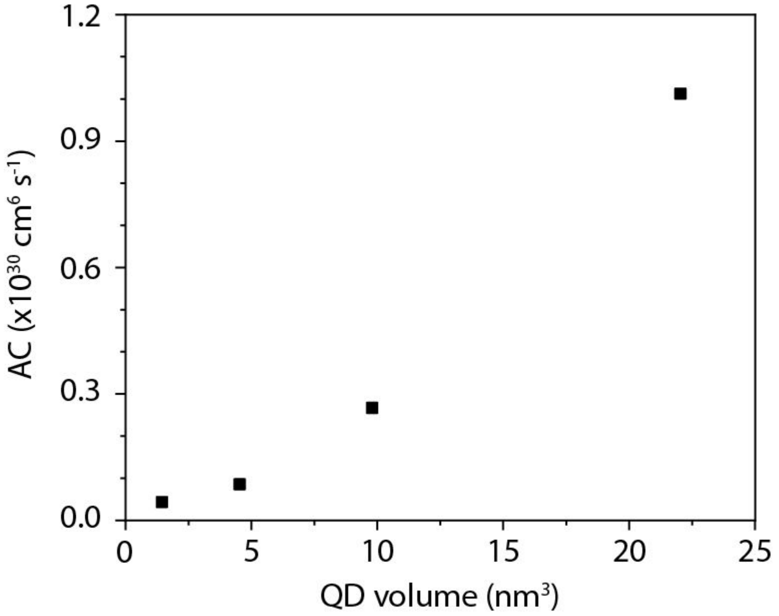

Figure S12 – Auger Constants of InP CQDs as a function of QD volume
